# Coping Style of Pigs Is Associated With Different Behavioral, Neurobiological and Immune Responses to Stressful Challenges

**DOI:** 10.3389/fnbeh.2019.00173

**Published:** 2019-08-01

**Authors:** Ellen Kanitz, Margret Tuchscherer, Winfried Otten, Armin Tuchscherer, Manuela Zebunke, Birger Puppe

**Affiliations:** ^1^Institute of Behavioural Physiology, Leibniz Institute for Farm Animal Biology (FBN), Dummerstorf, Germany; ^2^Institute of Genetics and Biometry, Leibniz Institute for Farm Animal Biology (FBN), Dummerstorf, Germany; ^3^Behavioural Sciences, Faculty of Agricultural and Environmental Sciences, University of Rostock, Rostock, Germany

**Keywords:** coping style, stress, limbic system, gene expression, immunity, domestic pig, welfare

## Abstract

Based on the animal’s reaction to environmental challenges, consistent but different coping styles can be identified, which in turn may have consequences for health and welfare. Therefore, profound knowledge of the complex interrelationships between individual behavioral response patterns, underlying neurobiological mechanisms and immunological effects is required. The aim of this study was to examine whether pigs with different coping styles exhibit distinct behavioral, neurobiological and immune responses to stressful situations. Therefore, pigs (*n* = 40) were classified as proactive, reactive or intermediate animals according to a repeatedly-performed backtest, and behavioral, neuroendocrine and immune alterations were analyzed without any stress before weaning on day 28 and after a stress treatment on day 32. Our results show that the behavioral responses in an open-field/novel-object test characterized proactive pigs as more active. There were no significant differences in adrenocorticotropic hormone and cortisol concentrations between pigs with different coping characteristics. However, we found that proactive pigs displayed significantly increased plasma noradrenaline levels in response to stress, which may reflect a higher sympathetic reactivity of these animals. Furthermore, the present study revealed coping style differences in mRNA expression of mineralocorticoid, glucocorticoid, oxytocin and arginine vasopressin receptors and the immediate early gene c-fos in stress-related brain regions. While proactive pigs responded to stress with higher mRNA expression of arginine vasopressin, mineralocorticoid and glucocorticoid receptors, reactive pigs displayed higher oxytocin receptor and c-fos mRNA expression, indicating different neurobiological mechanisms of distinct coping styles in response to stressful challenges. Moreover, we also found humoral immune differences between proactive, intermediate and reactive animals. Proactive pigs had a higher total serum IgA concentration before and after stress treatment, with a significant increase in response to stress compared to reactive and intermediate pigs. In contrast, stress-induced IgM concentrations only increased in reactive and intermediate animals, suggesting that the effects of coping style on humoral immunity may differ depending on the specific function of the immunoglobulin classes. In conclusion, this multidisciplinary study expands the concept of coping style in farm animals, particularly in terms of individual stress reactivity and disease susceptibility, and thus contributes to the understanding of the biology of animal welfare.

## Introduction

The concept of personality is used both in humans and a variety of wild and domestic animals and is referred to as a correlated set of individual behavioral and physiological traits that are consistent over time and contexts (reviewed by Finkemeier et al., 2018). The coping style is an individual adaptive strategy that describes the animal’s response to its environment in terms of reducing the effect of aversive stimuli (Folkman and Lazarus, 1988; Koolhaas et al., 1999), and therefore coping can be considered as one aspect of the personality concept (Finkemeier et al., 2018). Variation in coping with naturally occurring challenges determines the evolutionary fitness of individuals and is an important factor in the regulation of populations (Koolhaas, 2008). In general, different coping styles have been distinguished, ranging from a more proactive or active pattern to a more reactive or passive pattern (Henry and Stephens, 1977). Animals that are characterized by a proactive coping style tend to be bolder, more aggressive, dominant and less flexible to a changing environment. Physiologically, proactive animals display high sympathetic-adrenomedullary (SAM) reactivity and low or moderate hypothalamic-pituitary-adrenal (HPA) axis responsiveness to stressors, while reactive individuals exhibit opposite behavioral and endocrine patterns (Koolhaas et al., 1999, 2007). Furthermore, coping style seems to be an important factor in explaining individual variation in immunology and vulnerability to immune-mediated disease (Kavelaars et al., 1999; Kiecolt-Glaser et al., 2002; Segerstrom, 2003). Thus, there is evidence that in proactive, more aggressive animals with a higher risk of wounding and inflammation, a cellular immune response dominates, while in reactive, slowly exploring animals with a higher risk of infection, a humoral immune response is observed (Korte et al., 2005).

There is growing evidence that stress-coping behavior and its associated physiological activity are assisted by a basic circuitry of cortico-limbic brain areas, including the prefrontal cortex (PFC), amygdala, hippocampus and hypothalamus (de Boer et al., 2017). The functional characteristics of this basic circuitry are determined by a dynamic interplay of various key signaling molecules, such as glucocorticoids, monoamines and neuropeptides. In particular, the neuropeptides oxytocin (OXT) and arginine vasopressin (AVP) are implicated in inter-neural communication within these brain areas to modulate emotional, social-behavioral and physiological responses (Holmes et al., 2003). Most of the research on the neuroendocrine mechanisms of coping style was done in rodents (Koolhaas et al., 2010; de Boer et al., 2017). However, there are species-specific differences in the neurobiological development of stress systems, and this may have consequences for the animal’s ability to cope with stress (Kanitz et al., 2011).

Comparative research has shown that, despite domestication, genetic selection and inbreeding, farm animals also show consistent individual variation in response to stressful situations. In pigs, different coping styles affecting behavioral and physiological stress reactions were related to the dominance of the animals but were also influenced by the familiarity of animals and the success during a social confrontation test (Otten et al., 1997, 2002). Distinct coping styles have been demonstrated based on behavioral responses in a backtest, in which a piglet is placed in a forced supine position for 60 s and is monitored for struggling behavior (Hessing et al., 1993, 1994; Geverink et al., 2002; Bolhuis et al., 2005). Repeated applications of the backtest in combination with other behavioral tests (e.g., human approach, open-field) revealed moderate individual consistency in behavioral responses over time and across situations, which indicated a certain coping preference in pigs that may be modulated by the environment (Zebunke et al., 2015, 2017). Other studies in pigs demonstrated differences in HPA axis reactivity (Hessing et al., 1994; Ruis et al., 2000), immune response (Bolhuis et al., 2003; Reimert et al., 2014) and gene expression profiles related to immune defense and recovery (Oster et al., 2015) between proactive and reactive animals. It is known that the ability to cope with stressful situations is related to welfare and health in farm animals (Koolhaas and Van Reenen, 2016; Rauw et al., 2017).

The present study integrated coping style-dependent behavioral differences of pigs in response to stressful situations with associated alterations in their neurobiological and immune systems. The coping style of pigs was defined according to their behavior in a backtest as proactive, reactive or intermediate. We tested the hypothesis that these animals exhibit different coping strategies to stress, which are associated with different behavioral, neuroendocrine and immune activation profiles as measured by open-field behavior, stress hormones and immunocompetence, as well as mRNA expressions of corticosteroid and neuropeptide receptors in stress-related brain regions.

## Materials and Methods

### Animals and Experimental Procedures

The present study used a total of 40 German Landrace piglets derived from 29 sows, which had been bred and raised in the experimental pig unit of the Leibniz Institute for Farm Animal Biology (Dummerstorf, Germany). During the suckling period, sows and their piglets were housed in a loose farrowing pen (6 m^2^) with a water-heated lying area and with unrestricted access to food and water, and at a nearly constant room temperature (28 ± 1°C) with controlled lighting (12/12 h light/dark cycle). The experiments were performed in two consecutive replicates. For each replicate, at 28 days of age (just before weaning) 20 piglets were selected according to sex (females and non-castrated males, sex balanced) and their previously tested backtest behavior to describe their coping style (see below). We limited the number of full siblings as much as possible (maximum of 2 subjects from the same mother). Other selection criteria were a weight >5 kg and good health. Health status was visually assessed using the following criteria: regular growth rate, refusal to eat, consistency of feces, coughing, lameness, skin discoloration and swelling on the body or joints. The selected piglets were allocated randomly to two experimental groups: (1) the basal group without any stress treatment (blood and tissue sampling before weaning on day 28); (2) the stress group, which involved a potentially stressful weaning procedure on day 28 and an additional short-term isolation period during an open-field/novel-object test on day 32 (blood sampling on days 28 and 32, tissue sampling on day 32). The 10 piglets from different litters in the stress group were separated from the sow and littermates, grouped together and housed in a weaner pen (4.5 m^2^ with fully slatted plastic floors and a solid head area in the middle). Piglets were offered a commercial pellet diet from an automatic feeder. Food and water were provided *ad libitum*.

Blood samples were taken from each piglet while the animals were in a supine position by an anterior vena cava puncture (the whole procedure lasted 30 s). One part of each blood sample was collected into ice-cooled polypropylene tubes containing an EDTA solution, placed on ice, and subsequently centrifuged at 2,000 × *g* for 15 min at 4°C for the extraction of plasma, which was then stored at –20°C until analysis of the adrenocorticotropic hormone (ACTH), cortisol, tumor necrosis factor-α (TNF-α), and stored at –80°C until adrenaline and noradrenaline analyses. Another part of each blood sample was collected into glass tubes containing sodium heparin and was stored on ice until processing for mitogen-induced proliferation assays of peripheral blood mononuclear cells (PBMCs). Additionally, whole blood samples were allowed to clot for 4 h at room temperature and were centrifuged at 1,000 × *g* for 15 min at 4°C to obtain serum for analyses of the total protein and immunoglobulins IgG, IgA and IgM.

For gene expression analyses, 10 piglets of the basal group per replicate were euthanized immediately after blood sampling at day 28 of age with an intravenous injection of T61^®^ (embutramide/mebezonium iodide/tetracaine hydrochloride, Intervet, Unterschleißheim, Germany); the remaining 10 piglets of the stress group per replicate were euthanized after blood sampling at day 32 of age. The brains were quickly removed (<5 min), and the PFC, amygdala, hippocampus, and hypothalamus were dissected out of both brain hemispheres and were stored in RNAlater (Qiagen, Hilden, Germany) at −80°C until further processing. A stereotaxic atlas of the pig brain served as a reference (Félix et al., 1999).

### Backtest and Animal Selection

The backtest was adapted according to Hessing et al. (1993) and has already been described previously in detail by Zebunke et al. (2015). Briefly, the backtest was repeatedly performed at the ages of 5, 12, 19 and 26 days (time points 1–4). Each piglet was put on its back in a V-shaped cradle and was gently held in this supine position. The test lasted for 60 s and began as soon as the piglet was lying immobile. The latency until the first struggling attempt, the total duration and the total number of all struggling attempts (frequency) were measured. Afterward, the piglets were returned to their farrowing pens.

In each replicate, all piglets that were born were tested and classified as either high resisting (HR; proactive), IM or low resisting (LR; reactive) animals after the last backtest according to method LD1234, which is recommended and described in Zebunke et al. (2017). In detail, for each measure latency (L) and duration (D) of struggling were recorded at the four time points and the lower and upper quartiles were calculated across all piglets of each replicate. In the following analyses, the quartiles were used as cut-offs for classification. For each piglet, each measure was classified separately, which resulted in eight classifications per piglet. To decide which category each pig should be assigned to, we used the procedure described by Hessing et al. (1993); i.e., pigs that show a high/low response in more than 50% of the parameters with not more than one conflicting low/high response are classified as HR/LR. All other pigs are classified as IM. Based on this classification method, the 40 study piglets were classified as 13 HR, 16 LR and 11 IM animals, of which 7 HR, 9 LR and 4 IM were assigned to the basal group and 6 HR, 7 LR and 7 IM piglets were assigned to the stress group. The results of the backtest are shown in Supplementary Table S1.

### Open-Field/Novel-Object Test

As previously described (Kanitz et al., 2004; Puppe et al., 2007), the behavioral response of piglets was individually tested for 10 min in a square open-field arena (2.80 m × 2.80 m × 1.25 m) within a noise-reduced room using Observer version 10.0 (Noldus Information Technology, Wageningen, Netherlands). The test order of the piglets was randomized and the arena was washed down with soapy water between tests (Donald et al., 2011). For open-field/novel-object testing, each piglet was alone in the open-field arena during the first 5 min of observation; then, a novel-object (traffic cone) was lowered approximately 10 cm above the ground for another 5 min. The following behavioral variables were scored: locomotion (all forms of locomotor or exploratory activity), standing or sitting (all forms of motionless inactivity), escape attempts (trying to leave the open-field by jumping against the wall or by manipulative nosing of the wall), excretion (urination, defecation) and contact with the object (active touching and manipulation of the object with the snout). The duration, frequency and latency of each observed behavioral variable were analyzed.

### Endocrine and Immunological Measurements

ACTH concentrations were measured in duplicate in 200 μl plasma with a highly sensitive and specific two-site ELISA assay (DRG Instruments GmbH, Marburg, Germany) according to the manufacturer’s instructions. The assay has been previously validated with porcine plasma (Kanitz et al., 2014). The lowest level of ACTH that can be detected by this assay is 3.3 pg/ml, and the intra- and inter-assay coefficients of variation (CV) were 2.3% and 4.5%, respectively.

Plasma cortisol concentrations were analyzed in duplicate using a commercially available ELISA kit (DRG Instruments GmbH, Marburg, Germany) according to the manufacturer’s guidelines. The assay was validated for use with porcine plasma. Serial dilutions of two porcine plasma pools (>100 ng/ml and <20 ng/ml) with the provided diluent demonstrated parallelism to the standard curve. Recovery was conducted by addition of four cortisol standards (0, 5, 50, 100 ng/ml) to porcine plasma in a 20 μl reaction volume. Recovery levels ranged from 87% to 102%. The sensitivity of the assay was 3.4 ng/ml, and the intra- and inter-assay CV were 6.1% and 9.4%, respectively.

The TNF-α concentrations were analyzed in plasma samples using a commercially available pig ELISA kit (Biosource Invitrogen, Carlsbad, CA, USA) according to the manufacturer’s instructions. The sensitivity of the TNF-α assay was 3 pg/ml, and the intra- and inter-assay CV were 6.2% and 8.2%, respectively.

Plasma concentrations of adrenaline and noradrenaline were measured in duplicate using HPLC with electrochemical detection as described previously (Otten et al., 2013). The intra- and inter-assay CVs were 4.6% and 8.5% for adrenaline and 3.3% and 1.9% for noradrenaline, respectively.

The total protein content in serum was determined by the biuret-method (BioquantW Protein 110307; Merck, Darmstadt, Germany).

Serum concentrations of immunoglobulins IgG, IgA and IgM were analyzed by porcine-specific enzyme-linked immunosorbent assays (ELISAs) according to the manufacturer’s instructions (Bethyl, Laboratories Inc., Montgomery, TX, USA). The intra- and inter-assay CVs for the ELISAs were <5% and <10%, respectively (Tuchscherer et al., 2012).

PBMCs were isolated from heparinized blood by density gradient centrifugation, and the responses of lymphocytes to the T-cell-specific mitogen phytohemagglutinin (10 μg/ml, PHA) and the B-cell-specific mitogen lipopolysaccharide (12.5 mg/ml, LPS) were assessed in a cell proliferation/viability assay as previously described by Tuchscherer et al. (2016). The optical density (OD) was measured with a microplate reader (Dynatec, Denkendorf, Germany) at a test wavelength of 550 nm and a reference wavelength of 690 nm. The results were expressed as a proliferation index (PI), which was calculated as the ratio of the OD in the presence of mitogen to the OD in the absence of mitogen.

### RNA Isolation and Quantification of Transcripts

Total RNA was isolated from individual PFC, amygdala, hippocampus, and hypothalamus samples with RNeasy Lipid Tissue Kit (Qiagen, Hilden, Germany), as recommended by the supplier. The RNA was quantified in a NanoPhotometer™ (IMPLEN, München, Germany). RNA quality was monitored with the Experion™ Automated Electrophoresis System (BIO-RAD, München, Germany) according to the manufacturer’s protocol. All samples were classified in the acceptable quality category (7 < RNA quality indicator ≤ 10) as determined by an RNA quality indicator > 8.5 (10 = intact RNA, 1 = highly degraded RNA).

The mRNA expression of the *NR3C1* gene, encoding the glucocorticoid receptor (GR), of the *NR3C2* gene encoding the mineralocorticoid receptor (MR), of the *OXTR* gene encoding the oxytocin receptor (OXTR), of the *AVPR1*_a_ gene encoding the arginine vasopressin receptor 1_a_ (AVPR1_a_) and of the *C-FOS* gene was monitored using a reverse transcription (RT) with subsequent real-time polymerase chain reaction (PCR), as described previously (Löhrke et al., 2005; Kanitz et al., 2012). RT was carried out with 500 ng total RNA using an iScript cDNA synthesis kit (BIO-RAD, München, Germany) following the guidelines of the manufacturer. The resulting cDNA was amplified by real-time PCR (iCycler, BIO-RAD, München, Germany) using an iQ SYBR Green Supermix (BIO-RAD, München, Germany). One microliter of the RT reaction solution was added to 10 μl PCR mix primed with gene-specific oligonucleotides (TIB MOLBIOL, Berlin, Germany). Based on the published cDNA and gene sequences (GR: accession no. AY779185; MR: accession no. M36074; OXTR: accession no. X71796; AVPR1_a_: accession no. L25615; c-fos: accession no. AJ132510), the primers were designed to span a corresponding intron and to anneal between 60°C and 70°C. The following primer sequences were used: GR (forward, 5′-GTT CCA GAG AAC CCC AAG AGT TCA-3′; reverse, 5′-TCA AAG GTG CTT TGG TCT GTG GTA-3′), MR (forward, 5′-GTC TTC TTC AAA AGA GCC GTG GAA-3′; reverse, 5′-CTC CTC GTG GAG GCC TTT TAA CTT-3′), OXTR (forward, 5′-GTG CCT CAT TCT CTT CCT AGC TCT-3′; reverse, 5′-AGG TGA TAT CCC ACA GTA GCT GA-3′), AVPR1_a_ (forward, 5′-GAA GAT GAC TTT TGT GAT CGT GAC-3′; reverse, 5′-CTT TGA ACA CAG TCT TGA AGG AGA-3′) and c-fos (forward, 5′-GGG ACA GTC TCT CCT ACT ACC ACT-3′; reverse, 5′-GGT GAG GGG CTC TGG TCT-3′).

The PCR was carried out using a hot start (3 min, 95°C; 30 s, 60°C; 45 s, 70°C), 39 additional cycles (10 s, 95°C; 30 s, 60°C; 45 s, 70°C) and a final cycle of 10 s, 95°C; 30 s, 60°C; 7 min, 70°C, which corresponded to denaturation, annealing and elongation, respectively. The specificity of the products was assessed using a melting point analysis, which started at 60°C and elevated to 90°C (1°C per 10 s), and by using agarose gel electrophoresis (2%). The oligonucleotide structure was verified by sequencing in a subset of the experiments. The relative quantification was performed using the quantification module in CFX Manager Software™ version 2.1 (BIO-RAD, München, Germany) based on the PCR efficiency and crossing point deviation of an unknown sample vs. a calibrator and standardization by nonregulated reference genes (Pfaffl, 2001; Vandesompele et al., 2002). Data for mRNA expression of the investigated genes were presented as relative expression ratios normalized to *ACTB* (Beta-actin) and *TBP* (TATA-box binding protein) as endogenous reference genes, which were not affected by the fixed factors used in the statistical analysis.

### Statistical Analyses

Statistical analyses were performed using the SAS software for Windows, version 9.4 (Copyright, SAS Institute Inc., Cary, NC, USA). Descriptive statistics and tests for normality were calculated with the UNIVARIATE procedure of the Base SAS software.

The behavioral data duration and latency were approximately normal and could be evaluated by analysis of variance (ANOVA) using the MIXED procedure in SAS/STAT software. The count data were analyzed by a Poisson model using the GLIMMIX procedure in SAS/STAT software. The ANOVA and the Poisson model contained the fixed effects replication (1–2), coping style (HR, IM, LR) and sex (male, female). Sow was also included as a random effect.

Endocrine and immunological data could be considered as approximately normal and were analyzed by repeated measurement ANOVA using the MIXED procedure. The model contained the fixed effects replication (1–2), coping style (HR, IM, LR), stress treatment (stress by weaning and OF/NO test, no stress as basal control), sex (male, female) and coping style × stress treatment interaction. Sow was also included as a random effect. Repeated measures on the same piglet were taken into account by the repeated statement of the MIXED procedure using a compound symmetry structure of the block diagonal residual covariance matrix.

Gene expression data were approximately normal and could be evaluated by ANOVA using the MIXED procedure. The ANOVA model contained the fixed effects replication (1–2), coping style (HR, IM, LR), stress treatment (stress by weaning and OF/NO test, no stress as basal control), sex (male, female) and coping style × stress treatment interaction. Sow was also included as a random effect.

Sex had no significant effect on the considered behavioral, endocrine, immunological and gene expression data (*p* > 0.38) and therefore sex was removed from all final models. Additionally, least squares means (LS means) and their standard errors (SE) were computed for each fixed effect in the models described above and all pairwise differences between LS means were tested using the Bonferroni procedure. The Bonferroni procedure controls the experiment-wise error rate for LS means comparisons, and therefore do not require a preceding *F* test (Ryan, 1959). Bonferroni was not used as a “*post hoc*” test, because this procedure can find significant contrasts when the overall *F*-test is nonsignificant and, therefore, suffer a loss of power when used with a preliminary *F*-test. Significance was defined as *p* < 0.05.

## Results

### Open-Field/Novel-Object Behavior

The results of the ANOVA indicated that the coping style (HR, IM, LR) affected the behavioral response in an open-field/novel-object test (Table 1). There were significant effects of backtest classification on duration (*F*_(2,9)_ = 12.68, *p* < 0.01) and frequency (*F*_(2,9)_ = 6.80, *p* < 0.05) of escape attempts and on duration (*F*_(2,15)_ = 3.81, *p* < 0.05), frequency (*F*_(2,15)_ = 9.70, *p* < 0.01) and latency (*F*_(2,15)_ = 4.68, *p* < 0.05) of excretion reactions. The pairwise multiple comparisons of the LS means (Table 1) revealed a higher duration of escape attempts of HR pigs compared to IM (*p* < 0.05) and LR (*p* < 0.01) pigs, as well as a higher frequency of escape attempts of HR pigs compared to LR pigs (*p* < 0.05). Furthermore, the duration of excretion reactions was significantly lower in HR pigs than in LR pigs (*p* < 0.05), and the frequency of excretion was lower in HR pigs compared to IM and LR pigs (*p* < 0.01). In addition, HR pigs displayed a longer latency to excretion than did LR pigs (*p* < 0.05).

**Table 1:** Open-field/novel-object behavior of piglets with a high resisting (HR), intermediate (IM) and low resisting (LR) backtest classification.

	Coping style			*p*-value (*F*-test)
Behavior	HR	IM	LR	Coping style
**Locomotion**
Duration (s)	137.61 ± 13.32	151.17 ± 11.87	133.30 ± 11.46	0.547
Frequency (counts)	65.15 ± 6.61	64.23 ± 5.89	61.50 ± 5.69	0.903
Latency (s)	24.71 ± 12.56	24.32 ± 11.19	25.43 ± 10.81	0.997
**Standing**
Duration (s)	199.36 ± 29.75	207.87 ± 26.50	229.55 ± 25.61	0.719
Frequency (counts)	50.38 ± 5.86	58.11 ± 5.22	53.85 ± 5.04	0.643
Latency (s)	10.63 ± 8.42	9.93 ± 7.50	20.98 ± 7.25	0.504
**Escape attempts**
Duration (s)	15.25 ± 2.02^a,c^	4.21 ± 3.22^b^	2.82 ± 1.67^d^	0.002
Frequency (counts)	5.67 ± 0.88^a^	2.96 ± 1.40	1.50 ± 0.74^b^	0.015
Latency (s)	243.09 ± 73.02	303.99 ± 116.49	379.49 ± 60.96	0.396
**Excretion**
Duration (s)	23.23 ± 9.19^a^	45.81 ± 9.19	57.19 ± 7.82^b^	0.045
Frequency (counts)	2.40 ± 0.76^c^	7.26 ± 0.76^d^	6.06 ± 0.64^d^	0.002
Latency (s)	238.48 ± 31.73^a^	149.79 ± 1.73	108.44 ± 26.99^b^	0.026
**Object contact**
duration (s)	25.72 ± 6.95	24.46 ± 6.17	23.05 ± 6.53	0.963
Frequency (counts)	4.30 ± 1.03	5.13 ± 0.92	4.27 ± 0.97	0.766
Latency (s)	345.56 ± 15.92	350.75 ± 14.13	350.91 ± 14.96	0.966

*Data are expressed as least square means (LS means) ± standard errors (SE). Significant differences are indicated by different superscripts within a row (^a,b^*p* < 0.05; ^c,d^*p* < 0.01; Bonferroni procedure)*.

### Endocrine and Immunological Parameters

ANOVA revealed a significant main effect of coping style on noradrenaline (*F*_(2,22)_ = 5.04, *p* < 0.05) and IgA (*F*_(2,38)_ = 8.31, *p* < 0.01) concentrations. Furthermore, a significant effect of stress treatment was found for ACTH (*F*_(1,29)_ = 89.57, *p* < 0.001), cortisol (*F*_(1,23)_ = 644.23, *p* < 0.001), TNF-α (*F*_(1,33)_ = 8.82, *p* < 0.01), noradrenaline (*F*_(1,16)_ = 11.78, *p* < 0.01), total protein (*F*_(1,24)_ = 40.04, *p* < 0.001), IgA (*F*_(1,19)_ = 19.51, *p* < 0.001), IgG (*F*_(1,18)_ = 4.98, *p* < 0.05) and IgM (*F*_(1,21)_ = 18.71, *p* < 0.001) concentrations, as well as on the PI PHA (*F*_(1,23)_ = 9.47, *p* < 0.01) and the PI LPS (*F*_(1,53)_ = 5.11, *p* < 0.05). There was no significant effect of the coping style × stress treatment interaction on endocrine and immunological parameters (respective *p*-values in Table 2). As also shown in Table 2, the results of the Bonferroni procedure indicated higher ACTH (*p* < 0.001) and cortisol (*p* < 0.001) concentrations in pigs after stress in HR, IM and LR animals, whereas a significant increase in noradrenaline concentration on stress (*p* < 0.05) was found only in HR pigs. The noradrenaline response to stress treatment was significantly higher in HR animals than in LR animals (*p* < 0.01). The total protein level decreased in HR, IM and LR pigs after stress (*p* < 0.01), but there was a significant increase of the IgA concentration on stress in HR pigs and an increase of the IgM concentration in IM and LR pigs (*p* < 0.05). Moreover, HR pigs displayed both higher basal (*p* < 0.05) and stress (*p* < 0.01) levels of IgA compared to IM and LR pigs.

**Table 2:** Endocrine and immune parameters in piglets with a HR, IM and LR backtest classification before weaning on day 28 (basal) and after weaning and an open-field/novel-object test on day 32 (stress).

	Coping style			*p*-value (*F*-test)		
Parameters	HR	IM	LR	Coping style	Stress	Coping style × Stress
**ACTH (pg/ml)**
Basal	30.44 ± 9.15^A^	25.34 ± 10.77^A^	29.72 ± 8.46^A^	0.636	<0.001	0.464
Stress	113.46 ± 13.81^B^	106.41 ± 11.83^B^	91.68 ± 10.97^B^			
**Cortisol (ng/ml)**
Basal	16.85 ± 2.29^A^	20.09 ± 2.41^A^	18.77 ± 1.95^A^	0.934	<0.001	0.545
Stress	63.58 ± 3.16^B^	58.08 ± 2.75^B^	60.87 ± 2.52^B^			
**Adrenaline (ng/ml)**
Basal	0.56 ± 0.11	0.50 ± 0.12	0.57 ± 0.10	0.416	0.359	0.484
Stress	0.84 ± 0.18	0.65 ± 0.15	0.44 ± 0.14			
**Noradrenaline (ng/ml)**
Basal	1.55 ± 0.22^A^	1.07 ± 0.25	1.07 ± 0.20	0.015	0.003	0.531
Stress	2.61 ± 0.36^B,a^	2.03 ± 0.31	1.29 ± 0.28^b^			
**TNF-α (pg/ml)**
Basal	31.43 ± 13.37	23.14 ± 14.64	45.54 ± 12.07	0.898	0.005	0.515
Stress	76.94 ± 21.06	71.83 ± 17.99	47.79 ± 16.71			
**Total protein (mg/ml)**
Basal	63.81 ± 1.78^A^	61.15 ± 1.94^A^	58.22 ± 1.61^A^	0.063	<0.001	0.998
Stress	52.67 ± 2.87^B^	50.16 ± 2.42^B^	47.33 ± 2.26^B^			
**IgA (mg/ml)**
Basal	0.22 ± 0.02^A,a^	0.15 ± 0.02^b^	0.15 ± 0.01^b^	0.001	<0.001	0.463
Stress	0.27 ± 0.02^B,a^	0.19 ± 0.02^b^	0.18 ± 0.02^b^			
**IgG (mg/ml)**
Basal	5.67 ± 0.39	5.11 ± 0.44	5.47 ± 0.36	0.591	0.038	0.984
Stress	5.19 ± 0.52	4.55 ± 0.49	4.99 ± 0.43			
**IgM (mg/ml)**
Basal	0.97 ± 0.09	0.77 ± 0.09^A^	0.77 ± 0.08^A^	0.305	<0.001	0.856
Stress	1.16 ± 0.12	1.00 ± 0.11^B^	1.03 ± 0.09^B^			
**PBMC**
Basal	2.67 ± 0.32	2.48 ± 0.35	2.60 ± 0.29	0.774	0.802	0.471
Stress	2.20 ± 0.51	2.97 ± 0.44	2.36 ± 0.41			
**Proliferations index PHA**
Basal	3.55 ± 0.22	3.66 ± 0.24	3.22 ± 0.19	0.648	0.005	0.673
Stress	2.83 ± 0.35	2.81 ± 0.29	2.80 ± 0.28			
**Proliferations index LPS**
Basal	1.80 ± 0.13	2.04 ± 0.14	1.63 ± 0.12	0.484	0.027	0.461
Stress	1.48 ± 0.21	1.51 ± 0.18	1.56 ± 0.17			

*Data are expressed as LS means ± SE. Within a row, significant differences are indicated by different lower case superscript letters (at least *p* < 0.05; Bonferroni procedure). Within a classification group, different capital superscript letters indicate significant differences between basal and stress treatment values (at least *p* < 0.05; Bonferroni procedure)*.

### Brain Receptor Expression

#### PFC

**Figure 1:**
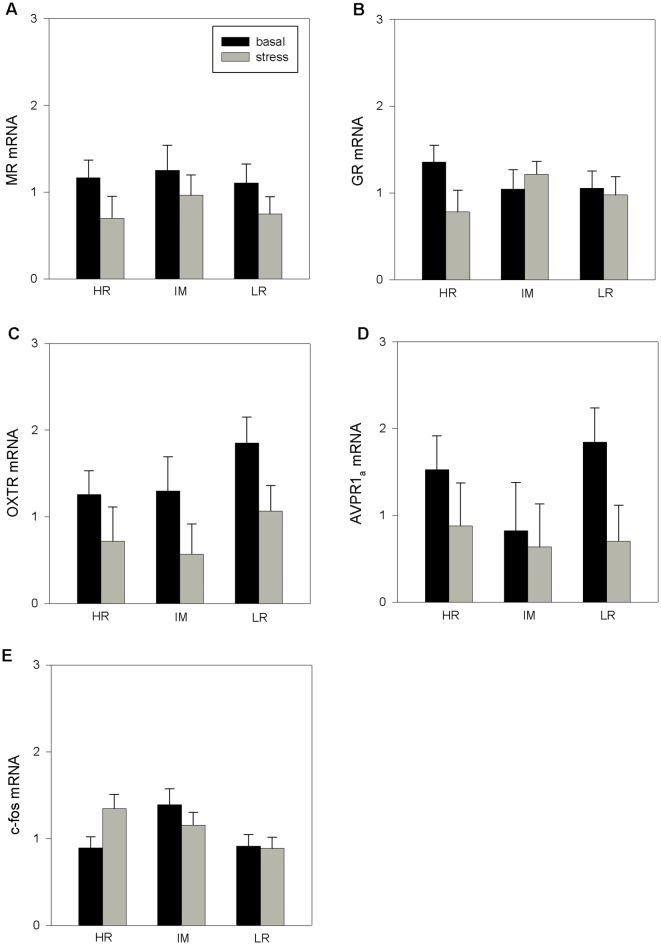
The basal and stress-induced mRNA expression of mineralocorticoid receptor (MR; **A**), glucocorticoid receptor (GR; **B**), oxytocin receptor (OXTR; **C**), arginine vasopressin receptor 1_a_ (AVPR1_a_; **D**) and c-fos **(E)** in the prefrontal cortex (PFC) of piglets with high-resisting (HR), intermediate (IM) and low-resisting (LR) backtest classifications. Data are expressed as arbitrary units after normalization to *ACTB* and *TBP* mRNA expression as endogenous reference genes and represent the least square means (LS means) ± standard errors (SE).

ANOVA indicated a main effect of coping style on c-fos mRNA expression (*F*_(2,32)_ = 3.26, *p* < 0.05). Furthermore, there was a main effect of stress treatment on OXTR mRNA expression (*F*_(1,28)_ = 6.21, *p* < 0.05) and a tendency for an effect of the stress treatment on MR mRNA (*F*_(1,31)_ = 3.80, *p* = 0.06) and AVPR1_a_ mRNA expression (*F*_(1,30)_ = 3.09, *p* = 0.08). The interaction of coping style × stress treatment had no significant effects on the mRNA expression that were investigated (MR: *p* = 0.929; GR: *p* = 0.581; OTXR: *p* = 0.929; AVPR1_a_: *p* = 0.588; c-fos: *p* = 0.682). The results of the Bonferroni procedure of the basal and stress-induced mRNA expression of MR, GR, OXTR, AVPR1_a_ and c-fos in the PFC of HR, IM and LR piglets are presented in Figure 1.

#### Amygdala

Statistical analyses revealed no significant effects of coping style (MR: *p* = 0.402; GR: *p* = 0.761; OTXR: *p* = 0.999; AVPR1_a_: *p* = 0.308; c-fos: *p* = 0.319), stress treatment (MR: *p* = 0.709; GR: *p* = 0.737; OTXR: *p* = 0.336; AVPR1_a_: *p* = 0.659; c-fos: *p* = 0.320) or interaction coping style × stress treatment (MR: *p* = 0.499; GR: *p* = 0.318; OTXR: *p* = 0.515; AVPR1_a_: *p* = 0.361; c-fos: *p* = 0.842) on the expression of MR mRNA, GR mRNA, OTXR mRNA, AVPR1_a_ mRNA and c-fos mRNA (data not shown).

#### Hippocampus

**Figure 2:**
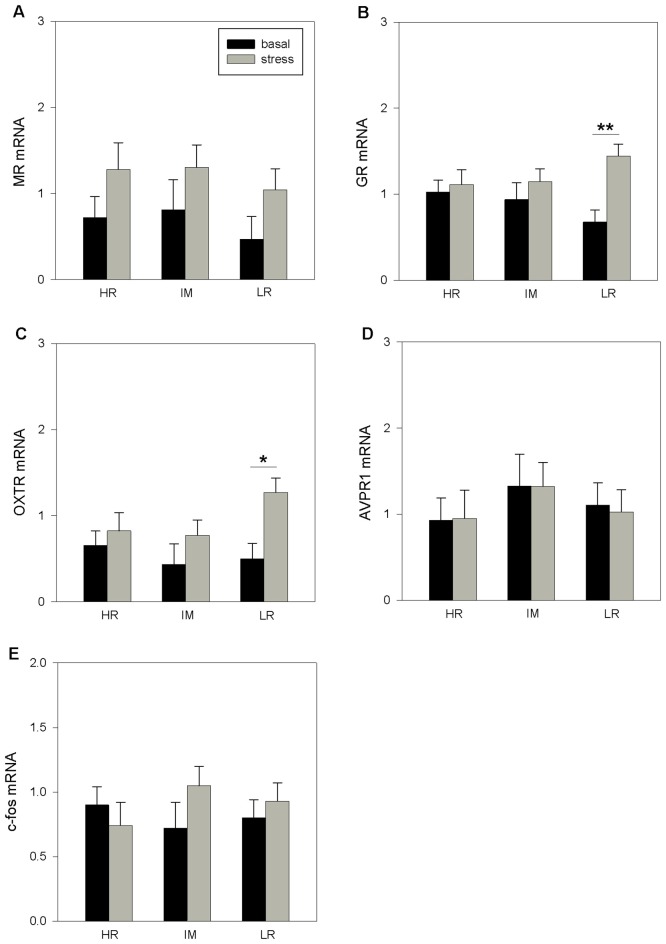
The basal and stress-induced mRNA expression of MR **(A)**, GR **(B)**, OXTR **(C)**, AVPR1_a_ **(D)** and c-fos **(E)** in the hippocampus of piglets with HR, IM and LR backtest classification. Data are expressed as arbitrary units after normalization to *ACTB* and *TBP* mRNA expression as endogenous reference genes and represent the LS means ± SE. Significant differences are indicated by asterisks (*p < 0.05, **p < 0.01; Bonferroni test).

There was no significant influence of coping style on the hippocampal mRNA expression (MR: *p* = 0.508; GR: *p* = 0.988; OTXR: *p* = 0.357; AVPR1_a_: *p* = 0.265; c-fos: *p* = 0.571). However, ANOVA indicated a significant main effect of stress treatment on MR mRNA (*F*_(1,32)_ = 5.68, *p* < 0.05), GR mRNA (*F*_(1,33)_ = 7.74, *p* < 0.01) and OXTR mRNA (*F*_(1,32)_ = 7.48, *p* < 0.05) expression. The interaction of coping style × stress treatment had no significant effects on the mRNA expression (MR: *p* = 0.987; GR: *p* = 0.261; OTXR: *p* = 0.244; AVPR1_a_: *p* = 0.778; c-fos: *p* = 0.271). The Bonferroni procedure (Figure 2) showed that stress treatment significantly increased the expression of GR mRNA (*p* < 0.01; Figure 2B) and OXTR mRNA (*p* < 0.05; Figure 2C) in LR pigs. There were no significant effects of stress treatment in HR or IM pigs (*p* > 0.1).

#### Hypothalamus

**Figure 3:**
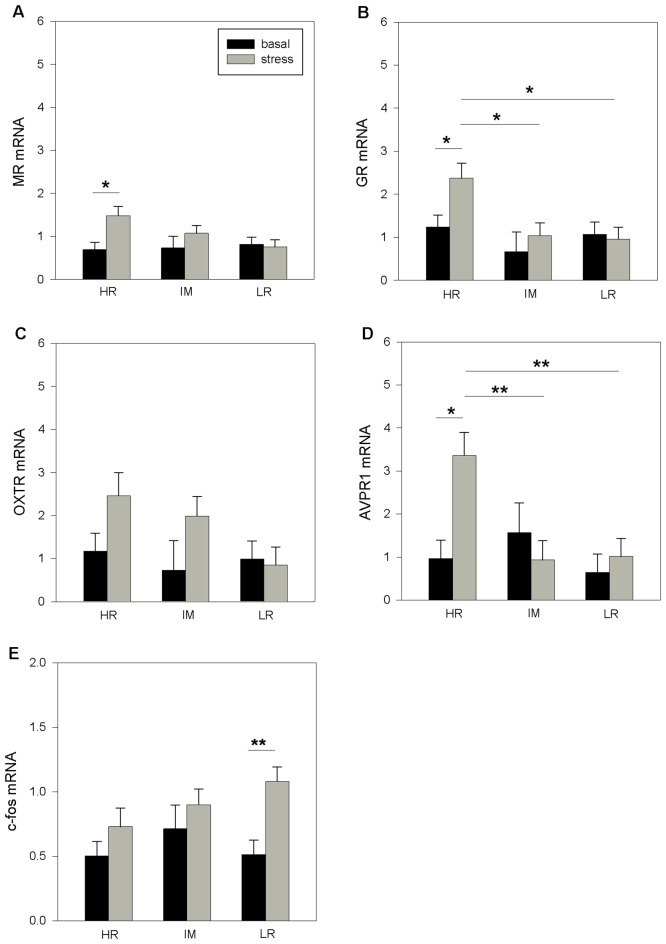
The basal and stress-induced mRNA expression of MR **(A)**, GR **(B)**, OXTR **(C)**, AVPR1_a_ **(D)** and c-fos **(E)** in the hypothalamus of piglets with HR, IM and LR backtest classification. Data are expressed as arbitrary units after normalization to *ACTB* and *TBP* mRNA expression as endogenous reference genes and represent the LS means ± SE. Significant differences are indicated by asterisks (*p < 0.05, **p < 0.01; Bonferroni test).

ANOVA revealed a main effect of coping style on GR mRNA (*F*_(2,32)_ = 4.93, *p* < 0.05), AVPR1_a_ mRNA (*F*_(2,32)_ = 4.42, *p* < 0.05) and c-fos mRNA (*F*_(2,32)_ = 4.192, *p* < 0.05) expressions. Furthermore, the statistical analyses indicated significant effects of stress treatment on MR mRNA (*F*_(1,32)_ = 4.91, *p* < 0.05) and c-fos mRNA (*F*_(1,32)_ = 7.92, *p* < 0.01) expression as well as a tendency for OXTR mRNA expression to be affected (*F*_(1,32)_ = 3.91, *p* = 0.06). A significant effect of the coping style × stress treatment interaction was found for AVPR1_a_ mRNA (*F*_(2,32)_ = 4.52, *p* < 0.05) expression. There were no further significant effects of the coping style × stress treatment interaction on the mRNA expression that were investigated (MR: *p* = 0.074; GR: *p* = 0.126; OTXR: *p* = 0.222; c-fos: *p* = 0.202). As shown in Figure 3, the Bonferroni procedure revealed a significant increase of MR mRNA (Figure 3A), GR mRNA (Figure 3B) and AVPR1_a_ (Figure 3D) mRNA expression in response to stress for HR pigs (all *p* < 0.05), while a stress-induced increase in c-fos mRNA was found only for LR pigs (*p* < 0.01; Figure 3E). Furthermore, the stress levels of GR mRNA and AVPR1_a_ mRNA in HR pigs were significantly higher than those in IM and LR pigs (*p* < 0.05).

## Discussion

The present study addresses the complex issues of behavior, neurobiology and immunity within the context of different stress coping strategies in domestic pigs. Our results demonstrate that individual coping styles of pigs, which were classified on the basis of HR (proactive), IM (intermediate) or LR (reactive) behavioral responses in a repeated backtest, revealed different behavioral outcomes in a combined open-field/novel-object test and showed distinct patterns of neuroendocrine and immune parameters after stressful challenges, as well as stress-related transcript levels in specific brain regions.

### Coping Behavior and Neuroendocrine Reactions

The behavioral responses of pigs in the open-field/novel-object test revealed that HR pigs exhibited more escape attempts and spent more time trying to leave the open-field than did IM and LR pigs. Furthermore, HR pigs displayed a lower duration and frequency of excretion reactions and a longer latency time to excretion. This indicated that HR animals showed more proactive behavior in this unpredictable challenging situation. Together with the backtest classification, the present behavioral measures that were taken during open-field exposure demonstrated consistency in pigs’ behavior across these different situations, which has also been shown for pigs in other studies (Ruis et al., 2000; Zebunke et al., 2017). Moreover, different behaviors in test situations have been assigned to different underlying motivations (Forkman et al., 2007). While locomotion and standing are considered to index general activity, escape attempts and excretion reactions are predominantly related to behavioral excitability in pigs (von Borell and Ladewig, 1992; Forkman et al., 2007; Puppe et al., 2007).

Before weaning, we found no differences in the ACTH and cortisol concentrations in pigs that were classified as HR, IM or LR animals. Although there was an increase in both hormones in response to stress challenges, such as weaning and short-term isolation during the open-field/novel-object test, there were no detected differences in the endocrine responses of the HPA axis between HR and LR pigs, which had already been described for both baseline and stress conditions in pigs (Ruis et al., 2000; Geverink et al., 2002). Indeed, studies in laboratory animals have shown that significant correlations between the HPA axis activity and coping style are not always found, and the correlations seem to depend on the type and duration of the stressor that is used (Koolhaas et al., 2007). With regard to the SAM system, HR pigs displayed significantly increased plasma noradrenaline levels in response to stress compared to IM and LR animals, which indicated a higher sympathetic reactivity of these animals. Studies in rats and mice have shown that during both social and non-social stressful situations, proactive animals reacted with a higher noradrenaline response in comparison with more reactive animals (de Boer et al., 1990; Sgoifo et al., 1996; Koolhaas et al., 2007). A recent study by our group also revealed a high sympathetic reactivity of proactive pigs in different behavioral contexts, as measured by heart rate and blood pressure (Krause et al., 2017), which supports the present findings. However, it should be noted that the activity of the sympathetic nervous system in general and the level of plasma noradrenaline, in particular, are considered as the metabolic and cardiovascular demands associated to physical activity, which could explain the positive correlation between proactive coping and sympathetic reactivity (Koolhaas et al., 2010).

### Coping and Neurobiological Substrates

The brain regulates the behavioral and physiological responses to a given stressor that may lead to successful adaptation or to pathophysiology and disease. In addition to the hypothalamus as the central stress response system, a distributed neuronal circuitry within the brain determines what is threatening and thus stressful to individuals (McEwen and Gianaros, 2010). Among the important regions in this stress circuitry system are the amygdala, which regulates emotional responses and emotional memories (Phelps and LeDoux, 2005), the hippocampus, which is involved in learning and memory processes (Sanders et al., 2003), and the PFC for decision making and executive control (McEwen and Morrison, 2013). Glucocorticoids, as end product of the HPA axis activation, are important mediators of these processes and exert their effects *via* two receptor subtypes, the MR and the GR. Both receptors are ligand-gated transcription factors that alter the expression of a wide variety of genes (de Kloet et al., 1998, 2005). In addition, AVP, OXT and their receptors are components of an integrated system that modulates social stress and social behavior (Landgraf and Neumann, 2004). Due to the widespread distribution of AVP and OXT receptors within the brain, central AVP and OXT concentrations can alter the neuronal activity in stress-related brain regions and may also be considered as neurobiological traits, which in concert may determine the appropriate physiological and behavioral responses to environmental challenges (Barberis and Tribollet, 1996; Hernando et al., 2001; de Boer et al., 2015). In general, AVP is associated with increased anxiety, arousal and activity, while OXT facilitates anxiety-reduction and affiliation, and imbalances in activity of both neuropeptides may be associated with mental disorders (Heinrichs et al., 2009).

The results of the present study demonstrate, for the first time in pigs, coping style differences in brain mRNA expression of MR, GR, OXTR, AVPR1_a_ and c-fos in response to stressful situations. There are only a few studies in different species that have investigated the expression of these receptors in relation to coping style. We found that HR pigs displayed an increase of MR, GR and AVPR1_a_ mRNA expression in the hypothalamus in response to stress. A higher post-stress expression of hypothalamic GR and MR mRNA was found in proactive fish compared to reactive fish, suggesting increased tolerance and stress response performance in these animals (Vindas et al., 2017). It is known that corticosteroid receptors may determine the sensitivity of the negative feedback of glucocorticoids on HPA activity (Myers et al., 2012). Regardless of coping style, previous studies in pigs have shown that social isolation stress caused an increase in GR mRNA expression in the hypothalamus, which may indicate enhanced feedback inhibition at this level (Kanitz et al., 2009; Tuchscherer et al., 2018). In addition to an increase in corticosteroid receptors in the hypothalamus, the stress treatment also caused higher mRNA expression of AVPR1_a_ in HR pigs. It is known that the expression of AVPR1_a_ can be affected by the GR and the interaction of these receptors may have context-specific effects on neural targets, influencing a variety of behaviors (Caldwell et al., 2017). Although no correlation analyses were performed because of the limited number of animals, the parallel increase in these receptors in HR pigs is an interesting result and indicates the AVPR1_a_ as an important part of the hormonal stress axis and coping style. With respect to coping style, most studies on AVP regulation were carried out in the context of male aggression. It was shown in several species of rodents that increased vasopressinergic activity in stress-regulated brain regions is associated with increased levels of aggressiveness and, in general, proactive coping styles (Veenema and Neumann, 2007; Koolhaas et al., 2010). As measured in microdialysates, an analysis of the AVP release in male intruder rats upon social confrontation revealed significant differences between an active and passive coping strategy; active intruders responded with increased intra-hypothalamic release of AVP, and passive intruders responded with decreased AVP release (Ebner et al., 2005b). Furthermore, micro-infusion of AVP into the hypothalamus enhances offensive aggression in male rats and hamsters (Winslow and Insel, 1993; Delville et al., 1996), whereas injection of an antagonist of the AVP V1_a_ receptor into the hypothalamus of male hamsters inhibits aggression (Potegal and Ferris, 1989). In addition, mutant mice with a deletion of the vasopressin receptor V1_b_ gene displayed no offensive aggressive (proactive) behavior anymore (Wersinger et al., 2002, 2007). Related to the present results, the applied stress treatments, including weaning and isolation during the open-field/novel-object test also revealed differences in the modulation of AVPR1_a_ between HR and LR pigs and indicated that the hypothalamus is a key region for the regulation of stress and the social environment.

Furthermore, in the hippocampus, only LR pigs displayed an increase in GR mRNA expression and additionally, an increase in OXTR mRNA expression in response to the stress treatment. There were also no differences in receptor gene expression before stress treatment in pigs classified as HR, IM or LR animals. As in our results, no differences were found in basal MR and GR mRNA expression in mice that were selected for short and long attack latency (Van Riel et al., 2002; Veenema et al., 2003). However, forced swimming for 5 min increased the MR mRNA expression in both mice lines in different hippocampal subfields (Veenema et al., 2003). With respect to brain oxytocin and the distinct modes of coping, intracerebral microdialysis experiments in rats showed that brain-released oxytocin modulates the behavioral response during forced swimming and promotes a passive stress-coping style (Ebner et al., 2005a). Pharmacological studies on rats have demonstrated an inverse relationship between aggressiveness and brain oxytocin, which indicates enhanced oxytocinergic activity with anti-aggressive effects in specific contexts (Calcagnoli et al., 2013, 2014). Similar to our results, weaning and an additional acute stressor in rats altered oxytocin receptor levels in limbic brain regions that are involved in emotional processing with potential modifications in brain function and social behavior (Farshim et al., 2016). Overall, the current results support the hypothesis that an endogenous balance between vasopressin and oxytocin signaling within the cortico-limbic circuitries may trigger the expression of proactive or reactive behavioral responses to stressors (de Boer et al., 2017).

Additionally, in the present study, we analyzed the expression of the immediate early gene c-fos as a molecular marker of neuronal activity. Studies on neuronal activation in laboratory animals using c-fos have identified distinct brain regions and cell types that are activated or inhibited during different stress-coping reactions (Walker et al., 2009; Clinton et al., 2011). Rats that responded in a social defeat paradigm with an active coping style exhibited less stress-induced c-fos expression than did rats with a passive coping style (Walker et al., 2009). Regarding our study, the data of the c-fos expression analyses revealed differences in neuronal processing between HR and LR pigs to stress with a significantly increased c-fos mRNA expression in LR pigs, which may indicate an anxious phenotype or a maladaptive response of these animals (Muigg et al., 2009).

### Coping and Immunity

In humans, personality and coping style are well characterized by differences in immunology and disease susceptibility (Cohen and Hamrick, 2003; Segerstrom, 2003; Dahl, 2010). There is also increasing evidence of coping style-related differences in stress reactivity and immunity in pigs (Schrama et al., 1997; Bolhuis et al., 2003; Reimert et al., 2014). For example, pigs with a proactive coping style had higher hemolytic activity of the complement system proteins to eliminate pathogens, and in an enriched environment these pigs had higher antibody titers in response to a novel antigen, keyhole limpet hemocyanin (KLH), than did pigs with a reactive coping style (Reimert et al., 2014). In the present study, we also found humoral immune differences between HR, IM and LR pigs. HR pigs had a higher total serum IgA concentration before weaning, and after weaning and an additional open-field/novel object test when compared to IM and LR animals. In addition, only HR pigs displayed an increase of serum IgA levels in response to the stress treatment. IgA is an important serum immunoglobulin that has the highest daily production rate of antibody classes, and the protective function of IgA is manifested through neutralization of pathogens and their toxins (Woof and Kerr, 2006). Moreover, a crucial role of naturally occurring serum IgA is to promote a strong anti-inflammatory effect mediated by its FcαRI receptor and to dampen excessive immune responses (Mkaddem et al., 2014; Monteiro, 2014). Proactive animals are known to show more aggressive and bolder behavior in stressful/challenging situations and have a higher risk of wounding and inflammation (Korte et al., 2005). Therefore, in the case of injuries that are caused by agonistic interactions, an elevated serum IgA level may be a functionally appropriate response to protect proactive animals and prevent the development of inflammatory reactions. Interestingly, the changes in serum IgA concentration covary with the noradrenaline response to the stress treatment in HR pigs, which is coherent with the bidirectional communication between the brain and the immune system *via* neuroendocrine pathways (Besedovsky and Rey, 2007; McEwen and Gianaros, 2010). The evolutionary explanation for this covariation is that spatio-temporal fluctuations in selection pressures favor different stress-coping strategies and immune outcomes under different environmental conditions (Cohen and Hamrick, 2003; Lazzaro and Little, 2009). Moreover, in our study, the serum IgM concentration was increased in LR and IM pigs after stress treatment. IgM antibodies participate as a first line of immune defense and their rapid synthesis ensures recognition and elimination of diverse types of infections (Racine and Winslow, 2009). Reactive animals, in turn, are non-aggressive, shy and show less active behavior in stressful situations. They are also at a higher risk of microbial infection compared with proactive animals (Korte et al., 2005). Therefore, a higher IgM level in response to stress can be considered immunologically useful for the protection of these animals against bacterial and viral infections. Hence, our findings indicate that both stress-induced increases in IgA concentrations in proactive pigs and increases in IgM concentrations in reactive pigs may be adaptive immune responses, which prepare organisms for the immunologic challenges that are most relevant for each behavioral phenotype. The effects of coping style on humoral immunity in pigs during stressful situations may differ depending on the special functions of differing immunoglobulin classes with regard to immunity.

In addition, the total serum protein concentration decreased in all classified pigs (HR, IM and LR) after the stress treatment, which is probably explained by the nutritional changes associated with the complex weaning process (Pluske et al., 1996; Le Dividich and Sève, 2000).

## Conclusion

The present study uses the concept of coping in farm animals as a tool to uncover the different neurobiological mechanisms underlying individual coping styles in response to stress. We demonstrated in a multidisciplinary approach that pigs classified as proactive, reactive or intermediate in a standardized backtest paradigm, displayed different coping strategies both in behavioral, neurobiological and immune responses to stressful challenges. Proactive coping in pigs is associated with more active behavior and increased noradrenergic activity. In terms of humoral immunity, proactive pigs exhibited a stress-induced increase in the serum IgA. By contrast reactive and intermediate animals responded with elevated IgM levels. The proactive pigs are characterized by increased gene expression of vasopressin receptors, whereas reactive pigs had a higher gene expression of oxytocin receptors in stress-related brain regions. In conclusion, coping styles represent a complex set of different physiological and behavioral options by which animals individually react to environmental challenges. The findings of this study expand our knowledge of the mechanisms of individual stress reactivity and disease susceptibility in animals, especially in confined environments.

## Ethics Statement

All procedures involving animal handling and treatment followed the regulations and guidelines of the German Animal Protection Law and were approved by the relevant authorities (Landesamt für Landwirtschaft, Lebensmittelsicherheit und Fischerei, Mecklenburg-Vorpommern, Germany; LALLF M-V/TSD/7221.3-1.1-016/11).

## Author Contributions

EK, MT and BP contributed to the conception and design of the study. EK, MT, WO and MZ performed the experiments and collected and analyzed the data. AT performed the statistical analyses. All authors interpreted the data. EK and MT wrote the manuscript.

## Conflict of Interest Statement

The authors declare that the research was conducted in the absence of any commercial or financial relationships that could be construed as a potential conflict of interest.
