# Supplementary material for: Coping Style of Pigs Is Associated With Different Behavioral, Neurobiological and Immune Responses to Stressful Challenges
Source: Front Behav Neurosci. 2019 Aug 1;13:173. doi: 10.3389/fnbeh.2019.00173 (PMC6686684; doi:10.3389/fnbeh.2019.00173)
Supplement: Supplementary file 1 [file Table_1.DOCX]

**SUPPLEMENTARY MATERIAL**

TABLE 1

Results of the backtest in both replicates (n1 = 152, n2 = 143).

|  | Replicate 1 | | | | | Replicate 2 | | | | |
| --- | --- | --- | --- | --- | --- | --- | --- | --- | --- | --- |
| Parameter | Mean | SD | Q25% | Median | Q75% | Mean | SD | Q25% | Median | Q75% |
| L1 | 21.3 | 16.7 | 9 | 16 | 25 | 23.6 | 18.4 | 9 | 16 | 34 |
| L2 | 32.2 | 18.6 | 16 | 27 | 50 | 35.3 | 19.5 | 18 | 31 | 60 |
| L3 | 17.6 | 15.1 | 8 | 12 | 22 | 32.6 | 18.9 | 17 | 28 | 51 |
| L4 | 35.1 | 18.3 | 18 | 32 | 57 | 34.7 | 20.5 | 16 | 33 | 60 |
| D1 | 12.7 | 9.7 | 5 | 11 | 18 | 13.2 | 9.5 | 6 | 12 | 19 |
| D2 | 9.9 | 9.4 | 2 | 8 | 15 | 8.7 | 8.6 | 0 | 7 | 15 |
| D3 | 15.3 | 8.4 | 9 | 16 | 21 | 11.6 | 10.1 | 3 | 9 | 19 |
| D4 | 9.3 | 8.9 | 1,5 | 7 | 16 | 10.3 | 10.1 | 0 | 9 | 16 |

Shown are the parameters latency until first struggle in all four repetitions (L1 – 4) and total duration of all struggling in all four repetitions (D1 – 4). Calculated are mean values (Mean) and standard deviation (SD) as well as the 25 % quartile (Q25%), the 50 % quartile (Median) and the 75 % quartile (Q75%) as a basis for the classification of the pigs in the coping style categories.
